# Spherical Body Protein 4 from *Babesia bigemina*: A Novel Gene That Contains Conserved B-Cell Epitopes and Induces Cross-Reactive Neutralizing Antibodies in *Babesia ovata*

**DOI:** 10.3390/pathogens12030495

**Published:** 2023-03-22

**Authors:** Juan Mosqueda, Diego Josimar Hernandez-Silva, Massaro W. Ueti, Adolfo Cruz-Reséndiz, Ricardo Marquez-Cervantez, Uriel Mauricio Valdez-Espinoza, Minh-Anh Dang-Trinh, Thu-Thuy Nguyen, Minerva Camacho-Nuez, Miguel Angel Mercado-Uriostegui, Gabriela Aguilar-Tipacamú, Juan Alberto Ramos-Aragon, Ruben Hernandez-Ortiz, Shin-ichiro Kawazu, Ikuo Igarashi

**Affiliations:** 1 Immunology and Vaccines Laboratory, C. A. Facultad de Ciencias Naturales, Universidad Autonoma de Queretaro, Carretera a Chichimequillas, Ejido Bolaños, Queretaro 76140, Mexico; 2 C.A. Salud Animal y Microbiologia Ambiental, Facultad de Ciencias Naturales, Universidad Autonoma de Queretaro, Av. de las Ciencias s/n Col Juriquilla, Queretaro 76230, Mexico; 3 Ph.D. Program in Biological Sciences, College of Natural Sciences, Autonomous University of Queretaro, Av. de las Ciencias s/n Col Juriquilla, Queretaro 76230, Mexico; 4 Animal Diseases Research Unit, Agricultural Research Service, US Department of Agriculture, Pullman, WA 99164, USA; 5 Veterinary Medicine Program, College of Natural Sciences, Autonomous University of Queretaro, Av. de las Ciencias s/n Col Juriquilla, Queretaro 76230, Mexico; 6 Master’s Program in Animal Health and Production, Facultad de Medicina Veterinaria y Zootecnia, Universidad Nacional Autonoma de Mexico, Av. Universidad 3000, Edificio A, Delegacion Coyoacan, Col. Ciudad Universitaria, Mexico City 04510, Mexico; 7 CENID-Salud Animal e Inocuidad/INIFAP, Carretera Federal Cuernavaca-Cuautla #8534, Col. Progreso, Jiutepec 62574, Mexico; 8 National Research Center for Protozoan Diseases, Obihiro University of Agriculture and Veterinary Medicine, Obihiro 080-8555, Japan; 9 Posgrado en Ciencias Genomicas, Universidad Autonoma de la Ciudad de Mexico, San Lorenzo 290, Esquina Roberto Gayol, Col. del Valle Sur, Delegacion Benito Juarez, Mexico City 03100, Mexico

**Keywords:** spherical body protein 4, *Babesia bigemina*, *Babesia ovata*, neutralizing antibodies

## Abstract

Bovine babesiosis is a tick-transmitted disease caused by intraerythrocytic protozoan parasites of the genus *Babesia*. Its main causative agents in the Americas are *Babesia bigemina* and *Babesia bovis,* while *Babesia ovata* affects cattle in Asia. All *Babesia* species secrete proteins stored in organelles of the apical complex, which are involved in all steps of the invasion process of vertebrate host cells. Unlike other apicomplexans, which have dense granules, babesia parasites instead have large, round intracellular organelles called spherical bodies. Evidence suggests that proteins from these organelles are released during the process of invading red blood cells, where spherical body proteins (SBPs) play an important role in cytoskeleton reorganization. In this study, we characterized the gene that encodes SBP4 in *B. bigemina*. This gene is transcribed and expressed in the erythrocytic stages of *B. bigemina*. The *sbp4* gene consists of 834 nucleotides without introns that encode a protein of 277 amino acids. In silico analysis predicted a signal peptide that is cleaved at residue 20, producing a 28.88-kDa protein. The presence of a signal peptide and the absence of transmembrane domains suggest that this protein is secreted. Importantly, when cattle were immunized with recombinant *B. bigemina* SBP4, antibodies identified *B. bigemina* and *B. ovata* merozoites according to confocal microscopy observations and were able to neutralize parasite multiplication in vitro for both species. Four peptides with predicted B-cell epitopes were identified to be conserved in 17 different isolates from six countries. Compared with the pre-immunization sera, antibodies against these conserved peptides reduced parasite invasion in vitro by 57%, 44%, 42%, and 38% for peptides 1, 2, 3, and 4, respectively (*p* < 0.05). Moreover, sera from cattle infected with *B. bigemina* cattle contained antibodies that recognized the individual peptides. All these results support the concept of *spb4* as a new gene in *B. bigemina* that should be considered a candidate for a vaccine to control bovine babesiosis.

## 1. Introduction

Bovine babesiosis is a tick-transmitted disease that is caused by intraerythrocytic protozoan parasites of the genus *Babesia* and characterized by clinical signs including fever, hemolytic anemia, jaundice, hemoglobinuria, and death [1] *Babesia bovis* and *Babesia bigemina* are the main causative agents of bovine babesiosis and are the only two species found in the Americas. Together with *Babesia ovata*, they infect cattle in Asia [1,2] and cause significant economic losses to the livestock industry [3]. An effective subunit vaccine against this disease is greatly needed.

During the lifecycle, the *Babesia* apical complex organelles produce and secrete proteins that are involved in red-blood-cell invasion during asexual reproduction in the vertebrate host [4,5]. Unlike other Apicomplexa parasites, which have dense granules, *Babesia* species have spherical bodies instead [6]. Since the first *B. bovis* spherical body protein (SBP) was discovered and characterized [7], evidence has suggested that the proteins from these organelles are released and translocated in the erythrocyte membrane, and at least in *B. bovis,* four SBPs designated as SBP1 to SBP4 are secreted during this process [8,9].

There is very little information about SBPs in other *Babesia* species. In *Toxoplasma*, several studies have shown that dense granule proteins are involved in the reorganization of the microtubular cytoskeleton network and the formation of the parasitophorous vacuole, and recently, the processes of parasite metabolism and nutrient intake have also been suggested [10,11,12,13,14]. Due to the differences between apicomplexan parasites, little is known about the spherical body organelles in *Babesia* species. *Babesia* members dissolve their parasitophorous vacuole right after invasion, and other spherical proteins are released during parasite invagination and before the invasion is complete [8]. This suggests that proteins from the spherical body organelle play an important role in cytoskeleton reorganization that helps to modify the cell membrane, which allows the intake of nutrients and the egress of the parasite after reproduction.

Conserved biological functions in secreted antigens could be used as targets for vaccine development or diagnosis. In this study, we describe the characterization of the *sbp4* gene in *B. bigemina*. We confirmed the gene transcription and expression, produced a recombinant protein, and used it to generate specific antibodies in cattle. We demonstrated that SPB4-specific antibodies identify SBP4 in *B. bigemina* and *B. ovata*. We also evaluated the sera of cattle that had been naturally infected with *B. bigemina* and evaluated them for the presence of specific antibodies against each peptide. Additionally, we evaluated cattle-specific SBP4 antisera for their capacity to reduce parasite multiplication of *B. bigemina* and *B. ovata* cultured in vitro. Finally, we designed peptides containing predicted B-cell epitopes that were conserved in 17 different isolates from six countries, generated antisera against each peptide, and evaluated them for their capacity to neutralize parasite invasion in vitro.

## 2. Materials and Methods

### 2.1. Bioethical Declarations

This study was approved by the Bioethics Committee of the Natural Sciences College of the Autonomous University of Queretaro (27FCN2016). The study was also approved by the Institutional Subcommittee for the Care of Animals in Experimentation under the master’s and doctorate program in Animal Production and Health Sciences of the National Autonomous University of Mexico (document: MC/2014-38).

### 2.2. B. bigemina DNA Isolation

*B. bigemina* DNA was obtained from infected ticks in different sources and geographical locations in Mexico: Veracruz (Acayucan) and San Luis Potosi (Tamasopo). DNA was also extracted from blood infected with *B. bigemina* collected from cattle in Mexico (Chiapas). Finally, erythrocytes infected with *B. bigemina* from the Mexico strain (Kuttler) were used to obtain DNA using a previously described method [14].

### 2.3. sbp4 Gene Amplification

To identify *B. bigemina* SBP4, a previously reported *sbp4* sequence from *B. bovis* was obtained from the NCBI database (accession number AF486507.1). Using the *B. bovis* sequence as a query, a BLAST analysis was performed on the database of the Sanger Institute against the *B. bigemina* reference genome from an Australian strain (https://www.sanger.ac.uk/resources/downloads/protozoa/babesia-bigemina.html, accessed on 15 May 2006) [15]. The sequence with the most significant similarity was obtained and analyzed using ORF Finder (https://www.ncbi.nlm.nih.gov/orffinder/, accessed on 15 May 2006) [16]. A set of primers was designed using Oligoanalyzer 3.1 [17]: Sbp4a-F: 5′-CAACAACTCAACGCAGTCAGG-3′ and Sbp4a-R: 5′-GCGCTAGTACGCGGAACAC-3′ amplifying outside the coding sequence an amplicon of 878 bp. Additionally, a forward primer (5′-CACCTTTGACAACGTCATCGAGGTGA-3′) and a reverse primer (5′-TCACTGGTGCTCCTCACCCTC -3′) were designed, and the CACC accessory sequence was included in the 5′ ends of the forward primer to allow directional cloning in posterior procedures.

Genomic DNA from each isolate and strain was used as a template to amplify the *sbp4* gene from *B. bigemina*. A double amplification program was used and consisted of a first single denaturation step of 95 °C for 1 min, followed by 10 cycles of 94 °C for 15 s, 54 °C for 30 s, and 72 °C for 50 s. A second amplification round was performed with the same conditions but a different annealing temperature (56.8 °C) for 20 cycles, followed by a final extension step at 72 °C for 7 min. PCR amplification was confirmed by DNA electrophoresis on 1% agarose gel. All PCR products were cloned in a pCR 4-TOPO vector according to the manufacturer’s instructions (Invitrogen^TM^, Thermo Fisher Scientific, Carlsbad, CA, USA). Four clones of each strain were sequenced by The Institute of Biotechnology, UNAM (Morelos, Mexico), and a consensus sequence was obtained from each isolate and strain.

### 2.4. Transcription Analysis

Transcription of *sbp4* was evaluated in blood stages. Intraerythrocytic parasites were obtained from whole blood and used for total RNA extraction with Trizol^®^ Reagent (Invitrogen, Carlsbad, CA, USA). The mRNA obtained was reverse transcribed using the Super ScriptTM II kit (Invitrogen, Carlsbad, CA, USA) according to the manufacturer’s instructions. The cDNA was amplified using the following primers: Sbp4b-F: 5′-GGCACAATGGTCGCCATT-3′ and Sbp4b-R: 5′-TCACTGGTGCTCCTCAACCTC-3′ amplifying an amplicon of 840 bp inside the coding sequence. The PCR protocol was used with an initial denaturation at 95 °C for 5 min, followed by 30 cycles consisting of denaturation at 95 °C for 15 s, annealing at 57 °C for 30 s, and extension at 72 °C for 1 min, followed by a final extension at 72 °C for 7 min. The amplicon was visualized on 1.8% agarose gel stained with ethidium bromide. The amplicon obtained was cloned using the TOPO^®^ TA Cloning^®^ kit (Invitrogen, Carlsbad, CA, USA) and transformed into *E. coli* TOP10 cells. Plasmid DNA was sent for commercial sequencing.

### 2.5. Cloning and Expression of sbp4 Gene

The PCR product of *sbp4* from the Chiapas strain of *B. bigemina* was directionally cloned in pENTR-D/TOPO entry vector following the manufacturer’s instructions (Invitrogen^TM^, Thermo Fisher Scientific, Carlsbad, CA, USA). Competent *E. coli* cells were transformed with recombinant DNA. Seven transformed colonies were analyzed by enzymatic restriction with NotI and AscI enzymes (Thermo Fisher Scientific, Carlsbad, CA, USA) to confirm the presence of the cloned *sbp4* gene. Once the *sbp4* entry clone was confirmed, subcloning was carried out by recombination in a pDEST-17 expression vector following the manufacturer’s instructions (Invitrogen^TM^, Thermo Fisher Scientific, Carlsbad, CA, USA). *sbp4*-pDEST-17 DNA was sequenced to confirm the proper insertion and recombination. The amino acid sequence was aligned to confirm the appropriate open reading frame position and gene length. An alignment was performed with the BLAST tool (https://blast.ncbi.nlm.nih.gov/Blast.cgi, accessed on 1 September 2012) to confirm the similarity to SBP4 of *B. bovis*.

The *E. coli* BL-21 AI strain (Invitrogen^TM^, Thermo Fisher Scientific, Carlsbad, CA, USA) was transformed with the *sbp4*-pDEST-17 construction, and four transformed colonies were analyzed by PCR using the *sbp4*-specific primers mentioned before. Once the transformation was confirmed, a pilot test of expression was carried out to assess the solubility of recombinant SBP4 protein (rSBP4) due to protein remaining in the insoluble fraction. Purification was performed as follows. One colony was chosen to set up a pre-inoculum culture that was incubated overnight at 37 °C at 225 rpm. After incubation, the cells were used to inoculate 500 mL of LB medium containing 100 µg/mL ampicillin. Three hours later, the optical density at 600 nm was measured, and once an O.D. of 0.4 was reached, 0.2% L-arabinose was added to the culture as an expression inducer.

The culture remained under the same incubation conditions for 3 h. The cells were centrifuged and washed three times with phosphate-buffered saline (PBS) at pH 7.4, and then cell disruption was performed by sonication at 80% amplitude with pulses of 10 s for 3 min. The cell lysate was centrifuged at 10,000× *g*, and the lysate pellet was used for protein purification under denaturing conditions by affinity chromatography with nickel beads following the manufacturer’s instructions (Ni-NTA superflow columns, Qiagen, Germany). Eluates were collected in individual tubes and analyzed by SDS-PAGE. Eluate 3 was used for the experiments as shown in Figure S1. Protein was quantified by the Bradford method and then used for cattle immunization as described in Section 2.8.

### 2.6. In Silico Analysis of Predicted SBP4 Protein Sequences

The predicted SBP4 protein sequences were analyzed in silico using 17 amino acid sequences of SBP4 (4 obtained in this work and 13 obtained from the Protein Database): AGC12840 (SP3, Salta, Argentina), AGC12832 (M1A Corrientes, Argentina), AGC12833 (M1P, Corrientes, Argentina), AGC12834 (M2P, Corrientes, Argentina), AGC12835 (M30 Corrientes, Argentina), AGC12836 (Mexico), AGC12838 (S2A, Salta, Argentina), AGC12839 (S2P, Salta, Argentina), AGC12829 (B38, Salta, Argentina), AGC12837 (S1A, Salta, Argentina), AGC12830 (Brazil), AGC12831 (Colombia), and the reference sequence from Australia (Bond strain) XP_012767973. A multiple-sequence alignment was performed using MUSCLE (https://www.ebi.ac.uk/Tools/msa/muscle/, accessed on 30 October 2013) [Edgar, 2014). The predicted amino acid sequences obtained were analyzed to (1) identify the presence and location of a signal peptide with the program SignalP 4.0 [18], (2) find functional domains and their localization with SMART [19], and (3) determine whether the predicted protein has transmembrane helices using TMHMM [20].

### 2.7. Search and Selection of Conserved B-Cell Epitopes in SBP4

The prediction of conserved B-cell epitopes was performed with different bioinformatic algorithms using the predicted sequence of the mature protein as a template. Hydrophobicity was determined with the Parker Method of the BcePred program (https://webs.iiitd.edu.in/raghava/bcepred/bcepred_team.html, accessed on 2 November 2013) [21], the Kolaskar Method algorithm of the BcePred program, and the Antigenic tool (http://www.bioinformatics.nl/cgi-bin/emboss/antigenic, accessed on 2 November 2013) [22]. Prediction of linear B-cell epitopes was performed using the ABCPred program (http://crdd.osdd.net/raghava/abcpred/, accessed on 2 November 2013) [23] and the Antibody epitope prediction tool of the IEDB server (http.//tools.immuneepitope.org/bcell/, accessed on 2 November 2013) [24].

A BLAST analysis was performed on the NCBI portal (https://blast.ncbi.nlm.nih.gov/Blast.cgi, accessed on 5 November 2013) and the Sanger Institute portal (https://www.sanger.ac.uk/action/BLAST, accessed on 5 November 2013) [15]. Four peptides with the best scores in the bioinformatic analyses and found specifically in *B. bigemina* SBP4 were selected. Predicted peptides were chemically synthesized as a multi-antigenic peptide system of eight branches (MAP8) by GL Biochem (Shanghai, China).

### 2.8. SBP4 Peptide and Anti-rSBP4 Antisera Production

The rSBP4 protein was purified by affinity chromatography and dialyzed. Two 7-month-old steers were immunized three times with 100 μg of rSBP4 emulsified with Montanide ISA 71 adjuvant (1:1) (Seppic, Courbevoie, France) as previously described (Hidalgo-Ruiz, Mejia-Lopez et al. 2022). Next, 8-week-old New Zealand rabbits were immunized four times with 100 μg of each individual SBP4 peptide suspended in PBS and emulsified with Montanide ISA 71 adjuvant (1:1) (Seppic, Courbevoie, France) as previously described (Mercado-Uriostegui, Castro-Sanchez et al. 2022). Two rabbits were immunized with each individual peptide subcutaneously at 14-day intervals. For cattle, each immunization was given subcutaneously at 21-day intervals.

Serum samples were obtained prior to each inoculation. A final bleed was done 10 days after the last immunization. Two rabbits and two cattle were administered the same adjuvant and PBS under the same immunization schedule, and their sera were used as controls. Sera were stored in small aliquots at −20 °C until use.

### 2.9. Expression Analysis of SBP4 in Merozoites

To evaluate the expression of SBP4, we first performed a western blot analysis (WB). Cattle sera containing specific anti-rSBP4 antibodies and control sera were diluted to 1:20 with PBS. Briefly, erythrocytes infected with *B. bigemina and B. ovata* (iRBC) were obtained from an in vitro culture with >4% parasitized erythrocytes. Lysates were prepared by washing the cells several times with ice-cold PBS until the supernatant was clear. Then, the pellet was frozen at –80 °C, thawed on ice, and washed again three times. All the centrifugations between washes were performed at 2500× *g* for 10 min. Finally, the pellet was thawed, the loading buffer was added, and after mixing, it was centrifuged briefly. The supernatant was used in SDS-PAGE (12%) at 100 V for 1 h. The proteins were transferred to a PVDF membrane at 100 V for 1 h using a semi-dry system. The membrane was blocked overnight at 4 °C with PBS containing 3% skim milk.

The membrane was incubated for 1 h at room temperature with each diluted antiserum and washed three times with agitation at room temperature for 15 min each in PBS and 0.1% Tween (PBS–T). The membrane was incubated under the same conditions using a protein G conjugated with HRP (Invitrogen, Frederick, MD, USA) diluted at 1:500 in TBS–T (0.1%) and skim milk, followed by a final wash. Finally, the reaction was visualized with ECL WB Detection Reagent (Thermo Fisher Scientific, Shinagawa, Tokyo, Japan) and analyzed in a chemiluminescent detection machine (ImageQuant LAS 500; GE, Electric, Tokyo, Japan).

To evaluate the expression pattern of SBP4 in intraerythrocytic merozoites, a confocal microscopy analysis was performed. Smears of *B. bigemina* and *B. ovata* culture in vitro were made on ProbeOn slides (Fisher Scientific, Ottawa, ON, Canada), fixed in methanol for 2 min, and stored at −80 °C for one day. Then, they were vacuum-dried and permeated at 4 °C for 60 min with acetone (90%) diluted with methanol. The cattle antisera were tested at a dilution of 1:50 and detected with a rabbit anti-bovine IgG coupled with Alexa Fluor–488 in PBS-T containing Hoechst 33342 for nuclei staining (Thermo Scientific, Waltham, MA, USA).

All the incubations were performed at 37 °C in a humidity chamber for 1 h. Between each incubation, 10 washes were done with PBS–T (0.2% Tween20), and the last washing step was done with distilled water. Serum from cattle immunized with PBS and adjuvant was used as a negative control. The slides were analyzed in a confocal microscope with lasers for Hoechst 33342, Alexa-488, and brightfield. A final merged image was obtained with the LAS Advanced Fluorescence software (Leica, Wetzlar, Germany).

### 2.10. In Vitro Neutralization Assays with Antibodies against rSBP4

To determine if anti-SBP4 antibodies could block the invasion process, a neutralization assay (NA) was carried out using *B. bigemina* and *B. ovata* cultured in vitro. First, the *B. bigemina* Argentina strain and wild-type *B. ovata* Miyake strain were cultured in a 96-well plate using a 200 µL total volume per well with 5% erythrocytes according to published protocols [25,26]. A pre-incubation step was carried out in an atmosphere of 5% CO_2_ for 30 min at 37 °C with a mix containing 60% media, 40% sera, and 1% iRBC with 1% parasitemia. Then, a culture medium with 4% nRBC was added, and, after a gentle mix, 200 µL of the sample was split between three wells.

The culture was maintained at 37 °C in a 5% CO_2_ atmosphere for 72 h with changes of media (120 µL of media plus 30 µL of sera) every 24 h. The control for the effect of the adjuvant (AC) was post-immunization serum from a rabbit immunized only with PBS plus adjuvant. At the end of the incubation, the percentage of parasitized erythrocytes was determined by counting 2000 erythrocytes in Giemsa-stained slides. For the statistical analysis, an ANOVA was performed followed by a Turkey test, and *p* values < 0.05 were considered significant. The data were analyzed with Prism GraphPad 9 Software.

### 2.11. Recognition of SBP4 Peptides by Antibodies from Cattle Naturally Infected with B. bigemina

We evaluated whether cattle exposed to *B. bigemina* generate circulating antibodies against conserved SBP4 peptides containing the predicted epitopes. First, 136 bovine sera samples were collected from tick-infested areas and tick-free areas: Aguascalientes Queretaro, Sinaloa, Veracruz, and Durango (all states in Mexico). The samples were analyzed by an indirect immunofluorescence antibody test (IFAT) following a reference protocol [WOAH, 2021]. The sera identified as “positive” were positive for *B. bigemina* antibodies and negative for *B. bovis* antibodies. The sera identified as “negative” were negative for both *B. bigemina* and *B. bovis* antibodies. There were 84 positive sera and 52 negative sera.

Selected positive and negative sera for the presence of *B. bigemina* antibodies were evaluated by an indirect ELISA as described elsewhere [27]. Briefly, the synthetic peptides were attached to a Corning 3590 polystyrene flat Bottom 96-well high-bind EIA/RIA Clear Microplate (Corning Inc., Corning, NY, USA) at a concentration of 10 µg/mL in carbonate/bicarbonate buffer (0.1 M sodium carbonate, 0.1 M sodium bicarbonate) and incubated overnight at 4 °C. The plates were washed three times with PBS-Tween 20 (PBS-T) 0.05% for 30 s and dried on an absorbent paper towel. Plates were blocked with 5% (*w*/*v*) skim milk in PBS-T for 1 h at 37 °C with shaking at 250 rpm. The washing process was repeated.

Each cattle serum was diluted to 1:40 in PBS-T 0.05% and incubated for 1 h at 37 °C with shaking at 250 rpm. The plates were washed and incubated with anti-bovine IgG (H + L) secondary antibody coupled to peroxidase (Jackson ImmunoResearch, Baltimore, MD, USA) at a 1:2500 dilution in PBS for 45 min at 37 °C. The plates were washed three times with PBS-T 0.05% for 30 s and dried on an absorbent paper towel. The signal was detected using 0.4 mg/mL OPD (Sigma-Aldrich, St. Louis, MO, USA) as a substrate.

After an incubation period of 20 min at room temperature, 100 µL of 2N sulfuric acid were added as a stop solution. The plates were read on an ELISA Microplate Reader (Bio-Rad, Hercules, CA, USA) at 450 nm. Each serum was analyzed in triplicate. Cutoff points were calculated using the negative reference control’s mean O.D. values. All sera with O.D. values above the cutoff point were considered positive for the presence of antibodies against *B. bigemina* SPB4, and all sera with O.D. values below the cutoff point were considered negative. The cutoff point was calculated as the mean + 3 standard deviations from a negative reference serum. The cutoff point was calculated for each peptide on each plate.

### 2.12. Neutralization Assays Using SBP4 Specific Peptides in B. bigemina Cultures In Vitro

Additional neutralization assays were performed to investigate whether antibodies against specific SBP4 conserved peptides had neutralizing capacity as reported previously for other *B. bigemina* antigens [28]. Briefly, a pre-inoculum of *B. bigemina* Puerto Rico strain was cultured, and when 6% parasitemia was reached the number of parasites was determined. Fresh medium supplemented with bovine red blood cells and serum was inoculated with about 1 × 10^6^ parasites contained in 16.5 µL. Triplicate assays were set up simultaneously for each anti-SBP4 antibody, and rabbit sera were added at a 1:5 proportion to each replicate.

To avoid interference with culture development due to the addition of rabbit sera, the proportion of rabbit serum in the culture was first evaluated, and the cultures were tested with a 1:5 serum proportion. The cultures were incubated at 37 °C in 5% CO_2_ atmosphere for 48 h. After incubation, the red blood cells were resuspended by pipetting up and down, and an aliquot of the homogenized culture was taken to prepare smears using glass slides. As controls, parasites were added to complete culture medium or serum from a rabbit immunized with adjuvant only. A mix of sera against all four peptides was also included.

Smears of each triplicate culture were stained with Giemsa. The percentage of parasitized erythrocytes (PPE) was determined by counting the infected and noninfected red blood cells in five representative fields. A student’s *t*-test was carried out for comparative analysis of means in nonpaired samples to test differences between the cultures. The data were analyzed using SPSS 22.0.

## 3. Results

### 3.1. B. bigemina sbp4 Gene Identification and Characterization

**Figure 1:**
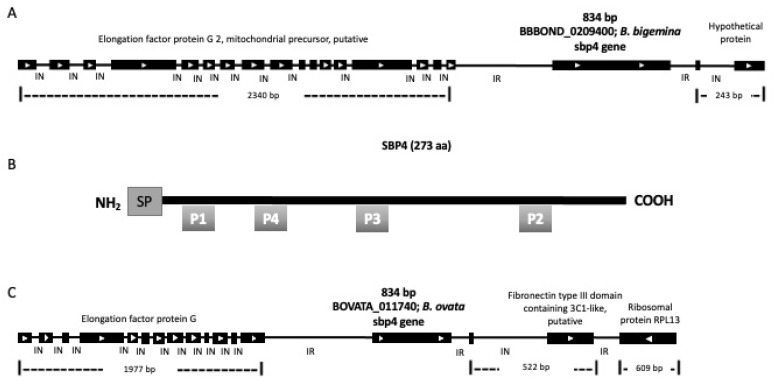
Bioinformatics analysis and genome location of *sbp4* in *B. bigemina and B. ovata*. (**A**) A sequence in GenBank (CDR95447.1) was identified in BBOND_0209400 locus of chromosome II. (**B**) Results of the bioinformatics analysis of the predicted SBP4 protein showing the signal peptide (SP) in the amino terminus, and the position of the predicted peptides 1–4 are indicated with grey boxes. (**C**) A sequence was identified in the BOVATA_011740 locus of *B. ovata*.

The results of BLAST analysis of the *B. bovis sbp4* gene in the *B. bigemina* genome database showed 56% identity with an ORF in Contig 3762 from the Sanger *B. bigemina* genome database (Figure 1A). When finally assembled and curated, according to the NCBI *B. bigemina* genome database, the hypothetical *sbp4* gene is in the BBOND_0209400 locus of chromosome II with accession number XP_012767973. The designed primers amplified a single band of 774 bp. This PCR product was cloned into a pCR 4.0 vector and transformed *sbp4* clones were sequenced.

The experimental full gene sequence of *sbp4* of each *B. bigemina* strain was obtained by sequencing. The *sbp4* consists of 834 nucleotides without introns that encode a protein of 277 amino acids. In silico analysis predicted a signal peptide that is cleaved at residue 20, producing a protein with a molecular weight of 28.88 kDa (Figure 1B, Supplementary Figures S1 and S2). No transmembrane or hydrophobic domains were predicted (Supplementary Figure S3). Together with the presence of a signal peptide, this analysis suggests that this protein is secreted.

The accession numbers for each sequence generated are as follows: Veracruz: OP079930.1, Chiapas: OP079929.1, Tamasopo: OP079928.1, Kuttler: OP079927.1. The predicted amino acid sequence alignment showed 98% identity among all strains (data not shown). These results demonstrated that the SBP4 protein is highly conserved among different *B. bigemina* strains.

Additionally, the BLAST analysis indicated that there was another sequence in the NCBI Protein database with a high percentage of similarity corresponding to *B. ovata* (accession number: XP_028865924.1). This sequence has 83% similarity, contains the same number of amino acids (277), including a signal peptide that is 20 amino acids long, and is encoded by a putative *sbp4* gene in the BOVATA_011740 locus in the *B. ovata* genome (Figure 1C).

### 3.2. SBP4 Is Transcribed and Expressed in B. bigemina Erythrocyte Stages

**Figure 2:**
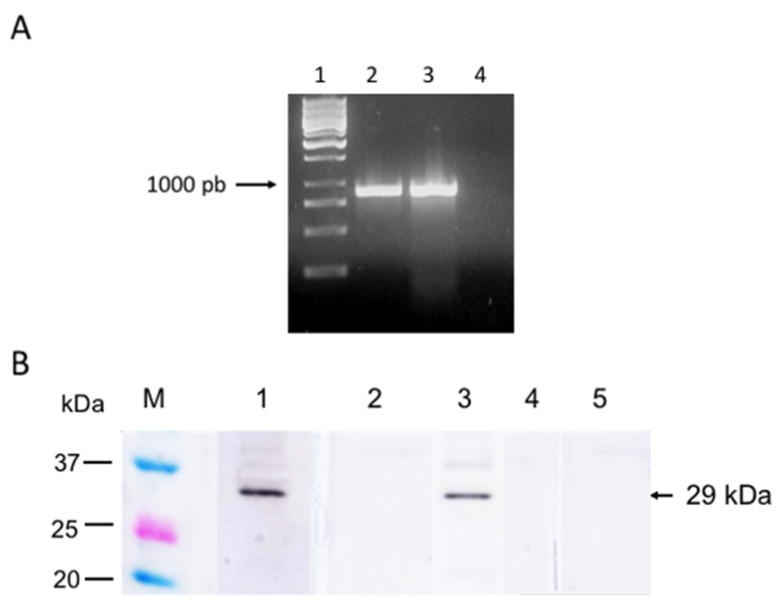
Transcription and expression analysis of SBP-4 by RT-PCR and western blot. (**A**) RT-PCR was visualized on a 1.8% agarose gel stained with ethidium bromide using primers to amplify an 840 bp amplicon. Lane 1: DNA ladder marker; Lane 2: *B. bigemina* cDNA with reverse transcriptase; Lane 3: *B. bigemina* DNA. Lane 4: *B. bigemina* cDNA without reverse transcriptase. (**B**) Western blot showing a specific band of approximately 29 kDa. Lane 1: proteins of *B. bigemina*-infected red blood cells incubated with post-immunization serum anti-rSPB-4; Lane 2: proteins of uninfected-RBC incubated with post-immunization serum anti-rSPB-4; Lane 3: proteins of *B. ovata*-infected red blood cells incubated with post-immunization sera anti-rSPB-4. Lane 4: proteins of *B. bigemina*-infected red blood cells incubated with control cattle serum (adjuvant only). Lane 5: proteins of *B. ovata*-infected red blood cells incubated with control cattle serum (adjuvant only). The molecular weight marker is shown in kDa (M).

To evaluate the expression, *B. bigemina sbp4* was first analyzed for transcription by RT-PCR. As shown in Figure 2A, cDNA of *B. bigemina* intra-erythrocytic parasites was amplified and showed a band of the expected size (840 bp) in agarose gel electrophoresis. The cDNA sequence obtained was 100% identical to the *B. bigemina sbp4* (accession number: XP_012767973, data not shown). No amplification was obtained when the mRNA sample was amplified without reverse transcriptase (Figure 2A, lane 4), indicating cDNA-specific amplification but not DNA. This confirms that the *sbp4* gene is transcribed in *B. bigemina* erythrocytic stages.

To confirm that SBP4 is expressed, a western blot was performed using red blood cells infected with *B. bigemina* using anti-*B. bigemina* rSBP4 bovine antibodies as primary antibodies (Figure 2B). A protein of approximately 29 kDa was detected in cells infected with *B. bigemina* (Figure 2B, lane 1). This experimental result corresponds with the predicted protein molecular weight in silico. No recognition was observed when uninfected red blood cell proteins were used (Figure 2B, lane 2) or when serum from cattle immunized with adjuvant alone was incubated with *B. bigemina*-infected red blood cells (Figure 2B, lane 5). Therefore, we showed that a *B. bigemina* protein of the same predicted molecular weight as SBP4 is expressed by erythrocytic parasites and detected by specific antibodies.

Additionally, the high similarity between *B. bigemina* and *B. ovata* SBP4 suggested the presence of conserved epitopes. To evaluate this, a western blot analysis of *B. ovata* infected-red blood cells using specific anti-*B. bigemina* rSBP4 antibodies showed the recognition of a specific band of the expected molecular weight (Figure 2B, lane 3). A serum from cattle immunized with only adjuvant was incubated with *B. ovata*-infected red blood cells and used as a negative control, and no band or signal was detected (Figure 2B, lane 4). These results indicate that the *B. ovata spb4* gene is expressed in erythrocytic parasites and is recognized by specific anti-*B. bigemina* SBP4 antibodies.

### 3.3. SBP4 Localizes to Round Organelles of B. bigemina and B. Ovata Merozoites and in the Cytoplasm of Infected Erythrocytes

**Figure 3:**
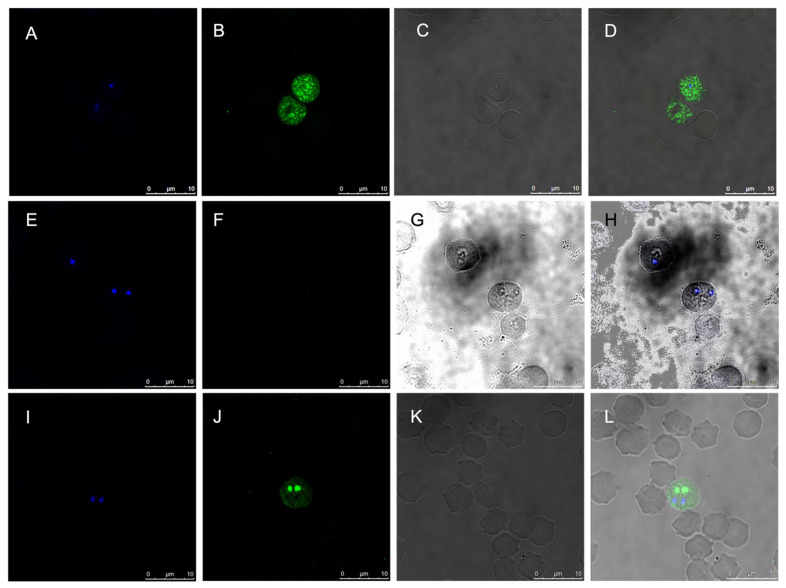
Analysis of the subcellular localization of SBP4 in merozoites of *B. bigemina* and *B. ovata.* Bovine antiserum against recombinant *B. bigemina* SBP4 was incubated with *B. bigemina* intraerythrocytic merozoites (**B**,**D**) or with *B. ovata* intraerythrocytic merozoites (**J**,**L**). No signal was observed when the serum of cattle immunized with adjuvant alone was used (**F**,**H**). Nuclei were stained with Hoechst 33342 (**A**,**E**,**I**). Bright field images (**C**,**G**,**K**) were also used to obtain merged images (**D**,**H**,**L**).

Once the gene product expression was confirmed, confocal microscopy was carried out to localize SBP4 in infected erythrocytes. Anti-rSBP4 antibodies bound to intraerythrocytic merozoites were detected with anti-bovine IgG antibodies coupled with Alexa Fluor-488 (Figure 3). There was a pattern consistent with the staining of a round organelle in the cytoplasm of the merozoites, as well as diffuse staining in the cytoplasm of the infected erythrocyte (Figure 3B,J). No staining was observed when merozoites were incubated with antibodies of negative control cattle vaccinated with adjuvant only (Figure 3F,H). Additionally, *B. ovata* infected erythrocytes were recognized by specific antibodies against *B. bigemina* SBP4 (Figure 3J,L). Merged images confirmed the localization of SBP4 to a round organelle adjacent to the nucleus of merozoites (stained with Hoechst 33342) and inside infected cells (Figure 3D,L).

### 3.4. Anti-SBP4 Antibodies Are Capable of Neutralizing B. bigemina and B. ovata’s Parasite Growth In Vitro

**Figure 4:**
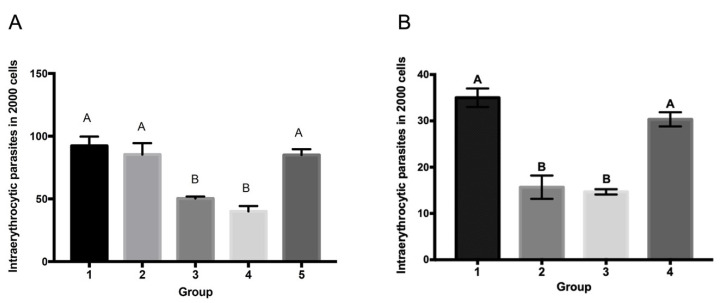
Antibodies against *B. bigemina* SBP4 neutralize growth of *B. bigemina* and *B. ovata* in vitro. (**A**) *B. bigemina* erythrocytic parasites maintained in vitro were cultured with (1) normal culture medium, (2) normal bovine serum, (3) serum of cattle one immunized with rSBP4, (4) serum of cattle two immunized with rSBP4, and (5) serum of cattle immunized with adjuvant. (**B**) *B. ovata* in vitro-maintained parasites were cultured with (1) normal culture medium, (2) serum of cattle one immunized with rSBP4, (3) serum of cattle two immunized with rSBP4, and (4) serum of cattle immunized with adjuvant. Statistical analysis was performed with an ANOVA and Tukey’s test using 95% confidence. Equal letters indicate no statistical differences, different letters indicate statistical difference. Bars indicate standard deviation.

To evaluate the capacity of antibodies against SBP4 to neutralize merozoite invasion, an in vitro neutralization assay was carried out. Cattle sera against rSBP4 reduced parasite growth in vitro compared to controls (Figure 4). Of a total of 2000 cells counted, the cultures incubated with two sera from cattle immunized with rSBP4 had mean intraerythrocytic parasite counts of 50.33 and 40.0, which are equivalent to 2.51% and 2.0%, respectively (Figure 4A). Controls including normal culture, culture with normal bovine serum, or culture with serum from an adjuvant-immunized bovine had mean parasite counts of 92.3 (4.61%), 85.33 (4.27%), and 85.0 (4.25%), respectively.

In terms of reduction of the percentage of intracellular parasites, the results for serum one were 54.4% (compared to the control medium), 58.98% (compared with the normal bovine serum), and 59.21% (compared with the adjuvant control serum). For serum two, the reduction in the percentage of intracellular parasites was 43.33% (compared to the control medium), 46.87% (compared to the normal bovine serum) and 47.05% (compared to the adjuvant control serum). There was a statistically significant difference (*p* < 0.05) between mean triplicate parasite counts in both cattle sera cultures and the three control group cultures (Figure 4). Interestingly, sera from cattle immunized with rSBP4B against *B. bigemina* also prevented the growth of *B. ovata* in vitro (Figure 4B). The two sera from cattle immunized with rSBP4 had mean intraerythrocytic parasite counts of 15.6 (0.78%) and 14.6 (0.73%), respectively, out of a total of 2000 cells counted. This was statistically significant (*p* < 0.05) compared to the mean count of 28.33 (1.42%) for parasitized erythrocytes in a total of 2000 cells in the normal culture and the count of 30.33 (1.52%) in the culture with bovine serum immunized with adjuvant. In terms of the reduction of the percentage of intracellular parasites, for serum one, the results were 55.06% (compared to control medium) and 51.43% (compared with adjuvant control serum). The reduction in the percentage of intracellular parasites for serum two was 51.53% (compared to the control medium) and 48.13% (compared to the adjuvant control serum).

### 3.5. SBP4 Peptides Containing Conserved Epitopes Are Recognized by Antibodies from Cattle Naturally Infected with B. bigemina

Bioinformatics analysis predicted the identification of four peptides that were specific for SBP4 in the *B. bigemina* proteome. Their position and sequence are shown in Table 1. All four peptides were conserved in the seventeen strains analyzed and were predicted to have B-cell epitopes. They were used for evaluating whether cattle infected with *B. bigemina* generate antibodies against SBP4. Strains were exposed to serum samples from *B. bigemina*-infected cattle and uninfected cattle. 136 bovine serum samples were analyzed (84 positive sera and 52 negative sera).

The results indicate that 81 out of 84 (96.42%) cattle serum samples contained antibodies against peptide one, 69 (82.14%) serum samples contained specific antibodies against peptide two, 80 (95.23%) cattle serum samples contained antibodies against peptide three, and all 84 (100%) serum samples contained specific antibodies against peptide four. As shown in Table 2, three cattle sera with antibodies against *B. bigemina* did not recognize peptide one, 21 positive cattle sera failed to recognize peptide two, four sera did not recognize peptide three, and one positive serum cattle failed to recognize peptide four. Additionally, five cattle sera that were negative by IFAT to *B. bigemina* recognized peptide one, six negative sera recognized peptide two, five negative sera recognized peptide three, and five negative sera recognized peptide four (Table 2).

### 3.6. Antibodies to SBP4 Peptides Containing Conserved Epitopes Neutralize B. bigemina Parasite Growth In Vitro

**Figure 5:**
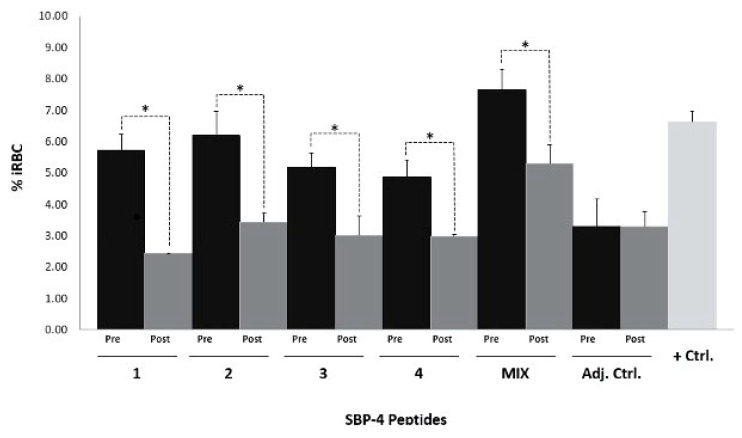
Antibodies anti-conserved *B. bigemina* SBP4 peptides neutralize growth of *B. bigemina* in vitro. *B. bigemina* erythrocytic parasites maintained in vitro were cultured with rabbit serum pre-immunization (Pre) or post-immunization (Post) against peptides one, two, three, four, or a mix of all four (MIX). As controls, cultures were added to the serums of rabbits immunized with adjuvant (Adj. Ctrl) or culture medium (+Ctrl). A student’s *t*-test was carried out for comparative analysis of means in nonpaired samples to test differences between the cultures. Asterisks indicate statistical differences. Bars indicate standard deviation.

Rabbit sera immunized with conserved SBP4 peptides were used to neutralize SBP4 in vitro. The anti-SBP4 peptide antibodies were capable of reducing parasitemia significantly in comparison with pre-immune rabbit sera (Figure 5). Antibodies against peptide one, peptide two, and peptide three were capable of reducing parasitemia by about 57%, 44%, of 42%, respectively, and the lowest inhibition percentage was given by anti-peptide four antibodies with 38%. The mixture of the sera against all the peptides reduced the parasite count by 33.65%, which was the lowest inhibition percentage (Figure 5). In parallel, *B. bigemina* cultures with pre-immune and post-immune rabbit serum immunized only with adjuvant were set up as controls, but the comparison of parasitemia percentage did not present a significant difference.

## 4. Discussion

Bovine babesiosis is caused by several *Babesia* species in areas of the world where ticks are endemic. All Apicomplexa parasites contain organelles that are necessary to invade and exit target cells. *Babesia* species possess spherical bodies that are equivalent to the dense granules in other genera, such as *Toxoplasma* or *Plasmodium*. The proteins of the spherical bodies are involved in important biological functions of the parasite, which makes them targets for the development of control measures such as vaccines.

By performing a BLAST search on the *B. bigemina* genome using the *B. bovis sbp2* gene as a query, we found a sequence with a high percentage of similarity and containing important features of a secreted protein, such as the presence of a signal peptide and the absence of transmembrane regions. Transcription and expression of the *sbp4* gene were demonstrated by RT-PCR and western blot, respectively. The results obtained from both experiments confirmed that *sbp4* is an active gene in the erythrocytic stages of *B. bigemina*, where it probably has a role.

We did not assess transcription and expression of *spb4* in other stages of the life cycle such as sexual stages or kinetes. However, no spherical bodies have been confirmed in the kinetes of *B. bigemina*. On the contrary, the presence of other organelles such as rhoptries was reported in kinetes [29], and the transcription of rhoptry proteins in *B. bigemina* kinetes has been confirmed [30]. This supports the idea that these organelles’ proteins might have different roles.

The confocal microscopy studies allowed us to confirm that SPB4 is expressed in the round organelles of merozoites and the cytoplasm of infected erythrocytes. This characteristic is also observed in other SBP4 homologues where an association with the cytoplasmic side of the infected erythrocyte membrane has been reported, including SBP4 in *B. bovis* and *B. orientalis* [31,32] Importantly, we also identified an *sbp4* homologue in *B. ovata,* where we demonstrated expression and a similar pattern of subcellular localization in infected erythrocytes. The high similarity (82%) between *B. bigemina* SBP4 and *B. ovata* SBP4 and the similar pattern of subcellular localization suggest a similar role of the protein in the biology of these species.

Specific bovine antibodies were produced against a recombinant SBP4 protein and utilized to evaluate their neutralization capacity in vitro. In the results, sera from cattle immunized with rSBP4 significantly reduced the percentage of intracellular parasites cultured in vitro. These results contrast with those obtained by Terkawi et al. for *B. bovis* SBP4, where no inhibition of in vitro growth of *B. bovis* cultures was observed in the presence of purified rabbit IgG against recombinant SBP4 [33]. In the *B. bovis* neutralization assay, purified rabbit IgG against a recombinant protein was used, while in the experiment, serum from cattle immunized with the recombinant SBP4 protein was used. However, inhibition of parasite growth in vitro was also observed when sera from bovines immunized with rSBP4 were incubated with *B. ovata*.

It should be noted that the similarity of the amino acid sequences between SBP4 of *B. bigemina* and *B. ovata* is 83%, which stands out with the similarity between the protein sequences of SBP4 of *B. bigemina* and that of *B. bovis*, which is only 21%. This low similarity could mean that *SBP4* from *B. bovis* is not homologous to SBP4 from *B. bigemina* or *B. ovata*. This would also imply that since these proteins do not share a similar sequence, they do not have the same function and do not participate in the same processes, so differences in the observed neutralization capacity could be expected.

An important characteristic of diagnostic or vaccine antigens is their ability to induce immune responses. By analyzing the sera from bovines that acquired the infection naturally in endemic areas of the disease, we were able to answer this question. We selected peptides that were conserved in all isolates and contained predicted B-cell epitopes. Between 82.14% and 100% of the sera analyzed contained antibodies that recognized at least one peptide.

Whether *B. bigemina* SBP4 can be used as a target antigen in a serological detection method remains to be determined. This has been indicated for *B. bovis* SBP4, which showed 85.3% concordance when it was utilized in an ELISA test to detect *B. bovis* antibodies [31]. It is likely that it will be necessary to evaluate whether it can discriminate between *B. bigemina* and *B. ovata* infection due to the high percentage of similarity in countries where *B. ovata* and *B. bigemina* are present.

Partial gene sequences of *B. bigemina sbp4* have been used in a multi-locus sequence typing (MLST) to discriminate strains in Argentina [34] due to a variable gene region, while the rest of the gene is highly conserved. However, the fact that the immune system recognizes conserved B-epitopes and generates antibodies against them in most cattle in the endemic areas tested supports the hypothesis that these conserved B-cell epitopes are immunogenic. These results are similar to those observed in other studies analyzing conserved B-cell epitope immunogenicity in other membrane-bound or organelle-secreted antigens in both *B. bigemina* and *B. bovis* [14,27,28,35,36,37]. This similarity suggests that those antigens are exposed to the immune system.

Finally, we tested the hypothesis that conserved peptides containing B-cell epitopes generate antibodies that neutralize the parasite’s invasion. The four peptides designed and tested generated antibodies that inhibited the in vitro growth of cultured parasites, and the highest inhibition was obtained with antisera against peptide one (57%), while the lowest was obtained with the antisera against peptide four (38%). However, when we mixed the sera from all peptides, we observed the lowest percentage inhibition. We have observed similar results when antibodies against different proteins are mixed and their neutralization capacity is tested, in which the reduction in parasite growth is not always greater than that of the individual antigen antisera [25]. This could be due to allosteric interference among specific antibodies, which varies in each antigen tested. However, this observation is not always true. When we mixed serum from each peptide and tested them together, the reduction in parasite counts was greater than that observed for individual peptides for different antigens [35,37]. Therefore, it is important to test antisera against each individual peptide and to determine its neutralizing ability. It might even be necessary to evaluate different mixtures of antibodies against different epitopes of different antigens to select the best combination in terms of neutralizing capacity.

In conclusion, we have demonstrated the presence of the *sbp4* gene in *B. bigemina*. This gene is transcribed and expressed in erythrocytic stages, and specific antibodies cross-react with *B. ovata.* Antibodies against recombinant *B. bigemina* SBP4 neutralize parasite growth in vitro for both species. Cattle naturally infected with *B. bigemina* produce antibodies specific for peptides containing conserved B-cell epitopes, and antibodies against conserved peptides neutralize parasite growth in vitro. All these findings support the inclusion of SBP4-specific peptides in a vaccine against *B. bigemina* and *B. ovata*.

## Figures and Tables

**Table 1:** Name, position, and sequence of designed SBP4 peptides. The peptides are ranked according to their predicted value as B-cell epitopes.

Name	Position in aa	Sequence
SBP4-1	51–70	MIHRQTDGCAPRTPVVYTPV
SBP4-2	215–234	KLVALIYHDVDGMKEALYHG
SBP4-3	145–163	KTIVVDINDVNDNKYLSYE
SBP4-4	71–89	RPNKLRHLVWSDTVIHGVG

**Table 2:** Analysis of the presence of antibodies in cattle naturally infected with *B. bigemina* to conserved SBP4 peptides by indirect ELISA.

		Peptide 1		Peptide 2		Peptide 3		Peptide 4	
Region	Farm	+	−	+	−	+	−	+	−
Aguascalientes	Villa Guadalupe	9	1	7	3	10	0	10	0
	Las Palomas	24	0	15	9	22	2	24	0
	Granja María I	4	0	2	2	3	1	4	0
Sinaloa	El Torito	7	0	6	1	7	0	7	0
	El Moral	10	0	8	2	10	0	10	0
	Herradura	1	0	1	0	1	0	1	0
	El Barón	2	0	1	1	2	0	2	0
Veracruz	La Esperanza	2	0	2	0	2	0	2	0
	San Faudila	2	2	4	0	4	0	4	0
	Las Torres	1	0	1	0	1	0	1	0
	Irineo Murillo	2	0	2	0	2	0	2	0
	Playa Vicente	2	0	1	1	2	0	2	0
	El Arbolito	2	0	1	1	2	0	2	0
	Manuel Antonio	1	0	1	0	1	0	1	0
	Buenos Aires	2	0	1	1	2	0	2	0
	El Orijuelo	9	0	9	0	8	1	9	0
Queretaro	Granja Araceli	1	0	1	0	1	0	1	0
	Amazcala	0	7	1	6	0	7	0	7
	Los Moreno	3	7	4	6	3	7	3	7
Durango	Arroyo Seco	2	33	1	34	2	33	2	33
Total		86	50	69	67	85	51	89	47
True positive		81		63		80		84	
False positve		5		6		5		5	
False negative		3		21		4		0	
True negative		47		46		47		47	

## Data Availability

The nucleotide sequence data reported in this article were submitted to the GenBank database under the accession numbers OP079930.1, OP079929.1, OP079928.1, and OP079927.1.

## Supplementary Materials

The following supporting information can be downloaded at: https://www.mdpi.com/article/10.3390/pathogens12030495/s1, Figure S1. SDS PAGE of the purified recombinant SBP4; Figure S2. Bioinformatics prediction of the signal peptide; Figure S3. Domain and signal peptide identification by SMART; Figure S4. Transmembrane domain identification.
