# Supplementary material for: Spherical Body Protein 4 from Babesia bigemina: A Novel Gene That Contains Conserved B-Cell Epitopes and Induces Cross-Reactive Neutralizing Antibodies in Babesia ovata
Source: Pathogens. 2023 Mar 22;12(3):495. doi: 10.3390/pathogens12030495 (PMC10051436; doi:10.3390/pathogens12030495)
Supplement: Supplementary file 1 [file pathogens-12-00495-s001.zip › pathogens-2261427-supplementary.pdf]

Supplementary Materials:

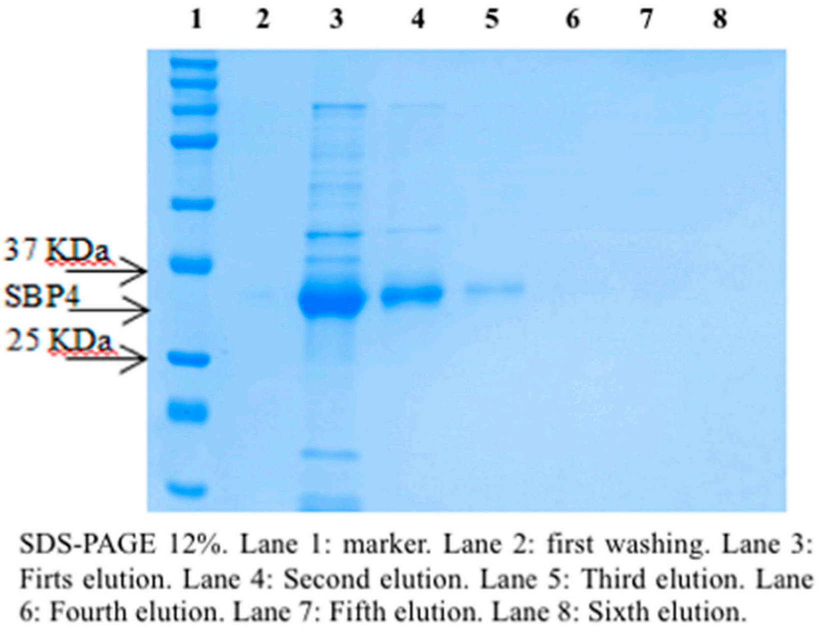

Figure S1. SDS PAGE of the purified recombinant SBP4.

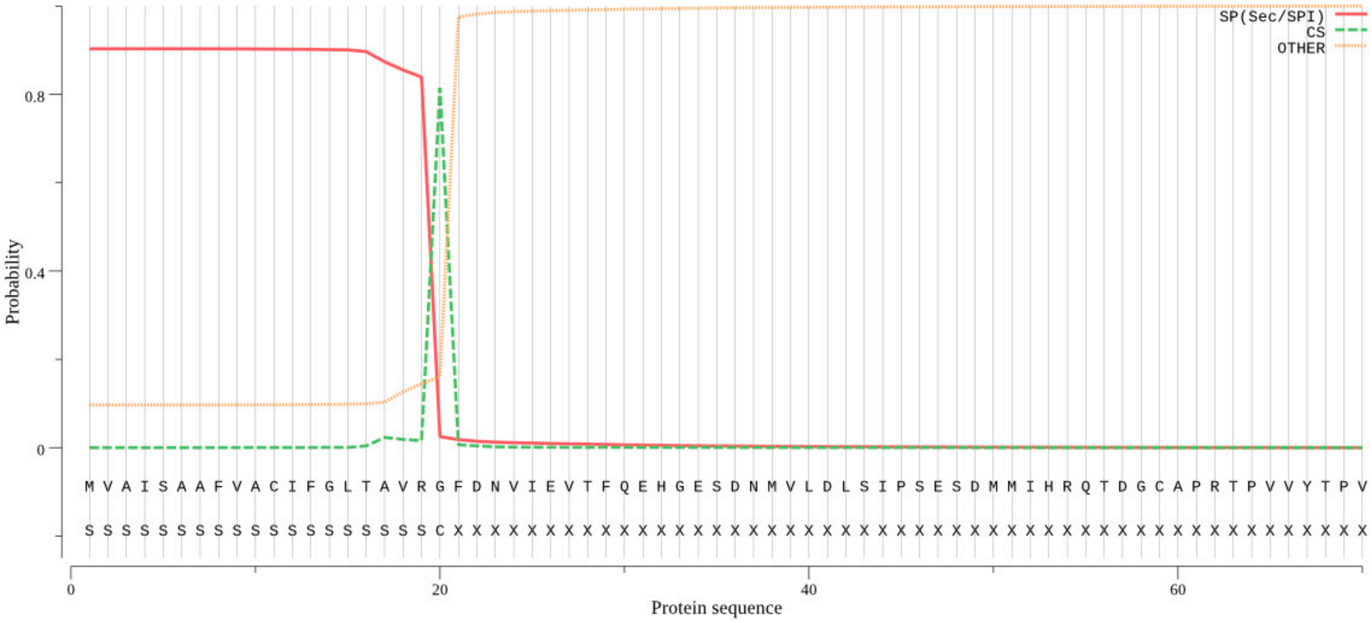

Figure S2. Bioinformatics prediction of the signal peptide.

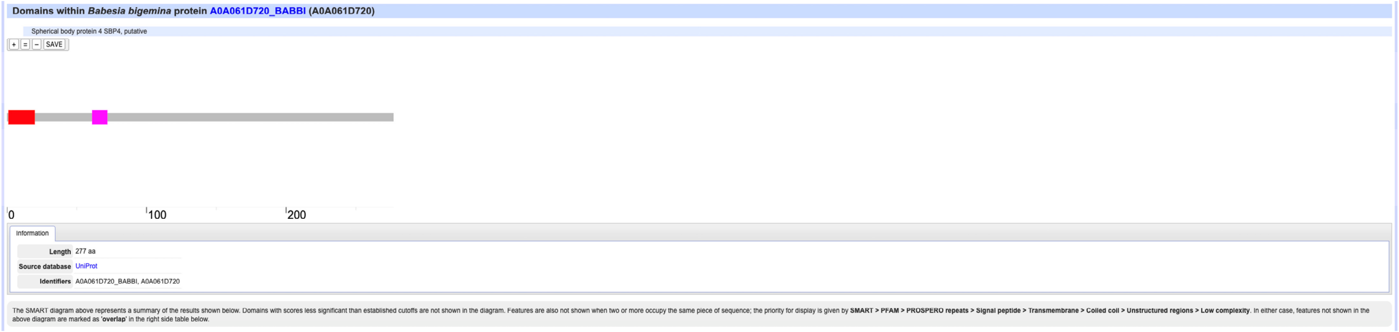

Figure S3. Domain and signal peptide identification by SMART.

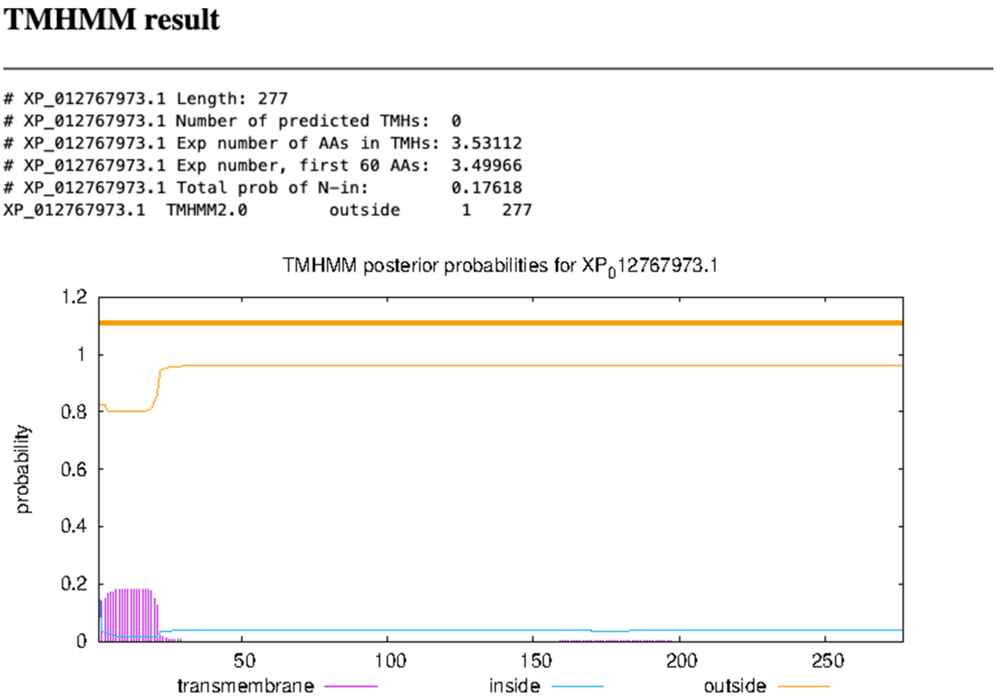

Figure S4. Transmembrane domain identification.
